# Comparison of β-blocker agents and mortality in maintenance hemodialysis patients: an international cohort study

**DOI:** 10.1093/ckj/sfae087

**Published:** 2024-03-27

**Authors:** Corey Toye, Manish M Sood, Ranjeeta Mallick, Ayub Akbari, Brian Bieber, Angelo Karaboyas, Murilo Guedes, Gregory L Hundemer

**Affiliations:** Department of Medicine, Division of Nephrology, University of Ottawa, Ottawa, ON, Canada; Department of Medicine, Division of Nephrology, University of Ottawa, Ottawa, ON, Canada; Clinical Epidemiology Program, Ottawa Hospital Research Institute, Ottawa, ON, Canada; Clinical Epidemiology Program, Ottawa Hospital Research Institute, Ottawa, ON, Canada; Department of Medicine, Division of Nephrology, University of Ottawa, Ottawa, ON, Canada; Clinical Epidemiology Program, Ottawa Hospital Research Institute, Ottawa, ON, Canada; Arbor Research Collaborative for Health, Ann Arbor, MI, USA; Arbor Research Collaborative for Health, Ann Arbor, MI, USA; Arbor Research Collaborative for Health, Ann Arbor, MI, USA; Department of Medicine, Division of Nephrology, University of Ottawa, Ottawa, ON, Canada; Clinical Epidemiology Program, Ottawa Hospital Research Institute, Ottawa, ON, Canada

**Keywords:** Beta blocker (β-blocker), cardiovascular, end-stage kidney disease (ESKD), hemodialysis, mortality

## Abstract

**Background:**

Despite a lack of clinical trial data, β-blockers are widely prescribed to dialysis patients. Whether specific β-blocker agents are associated with improved long-term outcomes compared with alternative β-blocker agents in the dialysis population remains uncertain.

**Methods:**

We analyzed data from an international cohort study of 10 125 patients on maintenance hemodialysis across 18 countries that were newly prescribed a β-blocker medication within the Dialysis Outcomes and Practice Patterns Study (DOPPS). The following β-blocker agents were compared: metoprolol, atenolol, bisoprolol and carvedilol. Multivariable Cox proportional hazards models were used to estimate the association between the newly prescribed β-blocker agent and all-cause mortality. Stratified analyses were performed on patients with and without a prior history of cardiovascular disease.

**Results:**

The mean (standard deviation) age in the cohort was 63 (15) years and 57% of participants were male. The most commonly prescribed β-blocker agent was metoprolol (49%), followed by carvedilol (29%), atenolol (11%) and bisoprolol (11%). Compared with metoprolol, atenolol {adjusted hazard ratio (HR) 0.77 [95% confidence interval (CI) 0.65–0.90]} was associated with a lower mortality risk. There was no difference in mortality risk with bisoprolol [adjusted HR 0.99 (95% CI 0.82–1.20)] or carvedilol [adjusted HR 0.95 (95% CI 0.82–1.09)] compared with metoprolol. These results were consistent upon stratification of patients by presence or absence of a prior history of cardiovascular disease.

**Conclusions:**

Among patients on maintenance hemodialysis who were newly prescribed β-blocker medications, atenolol was associated with the lowest mortality risk compared with alternative agents.

## Graphical Abstract

**Graphical Abstract:**
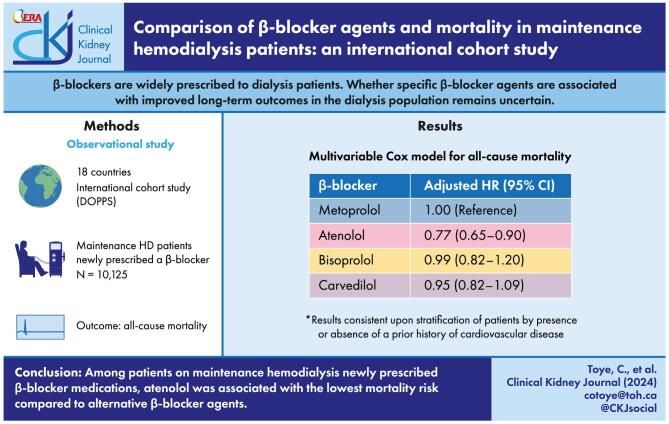

KEY LEARNING POINTS **What was known:**

- β-blockers are commonly prescribed to dialysis patients despite a lack of clinical trial data.
- Most prior observational studies indicate a survival benefit with β-blocker use in the dialysis population.
- Whether specific β-blocker agents (which display considerable heterogeneity in pharmacologic variables such as cardioselectivity and dialyzability) are associated with improved long-term outcomes compared with alternative β-blocker agents in the dialysis population remains uncertain.

**This study adds:**

- In a large international cohort of maintenance hemodialysis patients newly prescribed β-blockers, atenolol was associated with the lowest mortality risk compared with alternative β-blocker agents.
- These findings were consistent for hemodialysis patients both with and without a prior history of cardiovascular disease.

**Potential impact:**

- The findings from this study will serve to inform clinicians on β-blocker selection for the hemodialysis patient population.

## INTRODUCTION

Individuals with end-stage kidney disease (ESKD) who require dialysis are at exceptionally high risk for cardiovascular morbidity and mortality. In fact, they experience a rate of cardiovascular disease mortality that is up to 10- to 20-fold higher than that of the general population [1, 2]. Consequently, there is a pressing need for effective strategies to reduce cardiovascular risk among dialysis patients. β-blocker medications are well-established therapies proven to lower cardiovascular morbidity and mortality among high-risk patients, including those with heart failure and coronary artery disease [3–5]. However, dialysis patients were largely excluded from the landmark clinical trials that established the cardio-protective benefits of β-blockers [6]. Despite this limitation, approximately two-thirds of dialysis patients are routinely prescribed β-blockers. This may be attributed to the significant prevalence of comorbidities such as heart failure, coronary artery disease, atrial fibrillation and hypertension within the dialysis population [7].

While a few small randomized controlled trials have studied β-blockers in dialysis patients [8–10], the pursuit of larger scale trials have been deemed unfeasible due to recruitment challenges stemming from a perceived lack of clinical equipoise [11, 12]. As a result, the majority of evidence for β-blocker use in the dialysis population is derived from observational data with the majority of these studies indicating a survival benefit [13–17]. Nevertheless, the optimal β-blocker agent remains uncertain. This uncertainty arises from the considerable heterogeneity among β-blocker medications in terms of key pharmacologic variables such as cardioselectivity [18] and dialyzability [19]. Prior studies that grouped and compared β-blocker agents based on cardioselectivity versus non-cardioselectivity or high- versus low-dialyzability have yielded mixed, and often conflicting, results. This ambiguity may be due to confounding factors arising from the failure to fully account for pharmacologic differences within this class of medications [20–22]. To avoid this issue, subsequent studies have chosen to compare individual β-blocker agents head-to-head rather than simultaneously comparing multiple β-blocker agents. Consequently, the available evidence is limited to only a few agents used in clinical practice [23, 24].

Herein, we investigated a large international cohort of maintenance hemodialysis patients newly prescribed β-blocker medications to study the association between individual β-blocker agents and all-cause mortality.

## MATERIALS AND METHODS

### Study design

We analyzed data from a large international observational cohort study of maintenance in-center hemodialysis patients prescribed β-blocker medications to compare mortality risk by β-blocker agent prescribed. This study was approved by the Ottawa Health Science Network Research Ethics Board (# 20 210 145-01H).

### Data source and study cohort

The Dialysis Outcomes and Practice Patterns Study (DOPPS) is an international prospective cohort study that collects information on adults (≥18 years of age) on maintenance in-center hemodialysis [25]. The current study included patients who participated in DOPPS phases 1–6 (1996–2018). DOPPS employs a two-stage stratified random cluster sampling process where facilities are randomly sampled within country-specific strata followed by random selection of 20–40 patients within each hemodialysis facility. DOPPS collects detailed information on individual patient-level data including demographics, comorbidities, medications, laboratory values, dialysis characteristics, hospitalizations and mortality. All variables are collected using uniform and standardized data collection tools. The disposition of all patients at participating centers (including death, transfer and kidney transplantation) are tracked. The current study included DOPPS participants from across 18 countries that were newly prescribed a β-blocker medication within the DOPPS study period. Participants were excluded for the following reasons: no follow-up data available, β-blocker start/end date not available, survival status unknown, DOPPS country with <50 participants total, individual already prescribed β-blockers at DOPPS entry and individual β-blocker agents with <1000 total users. Further details on the DOPPS study design and methodology have previously been described [25].

### Exposure

The exposure of interest was the individual β-blocker agent prescribed. We included only β-blockers prescribed for at least 1000 DOPPS participants. With this criterion, the following four β-blocker agents were included: atenolol, bisoprolol, carvedilol and metoprolol. We only included participants who were newly prescribed a β-blocker medication within the DOPPS study period.

### Outcome

The primary study outcome was all-cause mortality.

### Statistical analysis

For baseline data, continuous variables were expressed as mean [standard deviation (SD)] if normally distributed and as median [25th–75th percentile interquartile range (IQR)] if non-normally distributed, while categorical variables were expressed as numbers (%). We examined the association between β-blocker agent prescribed and all-cause mortality by using Cox proportional hazards models to estimate crude and adjusted hazard ratios (HR) along with 95% confidence intervals (CI) based on an “intention-to-treat” design (i.e. a patient was categorized based on the original β-blocker agent even if later switched to a different agent). Metoprolol served as the reference β-blocker agent for comparison as it was the most commonly prescribed β-blocker in the cohort. The adjusted HRs were determined via Cox proportional hazards models adjusting for the following variables selected *a priori*: age, sex, race, body mass index, systolic blood pressure, diastolic blood pressure, dialysis-specific characteristics (vintage, sessions per week, treatment time and single-pool Kt/V), comorbidities [coronary artery disease, myocardial infarction, coronary artery bypass graft surgery, heart failure, atrial fibrillation, pacemaker, stroke, hypertension, peripheral vascular disease, diabetes mellitus and cancer (not including skin cancers)], statin use, clopidogrel use and year of β-blocker initiation. Each of these variables had <15% missingness; the missing values were imputed using the predictive mean matching method for continuous variables and discriminant function method for categorical variables [26]. Additionally, the Cox models accounted for clustering by country. Study index date was defined as the date at which the DOPPS participant was initially prescribed a β-blocker. Withdrawal from DOPPS and conclusion of the study period were considered censoring events. A secondary analysis was performed stratifying the participants based on whether they had a prior history of cardiovascular disease (defined as coronary artery disease, myocardial infarction, coronary artery bypass graft, heart failure, atrial fibrillation or pacemaker) or not. Sensitivity analyses were conducted whereby: (i) blood pressure was not adjusted for within the regression models, (ii) participants were stratified by age <65 years or ≥65 years, (iii) participants were stratified by whether they had a prior history of heart failure or not and (iv) an “as treated” study design was employed whereby participants were further censored if the β-blocker agent they were prescribed was discontinued. All statistical analyses were performed using SAS version 9.4 (SAS Institute Inc., Cary, NC, USA).

## RESULTS

### Baseline characteristics

**Figure 1:**
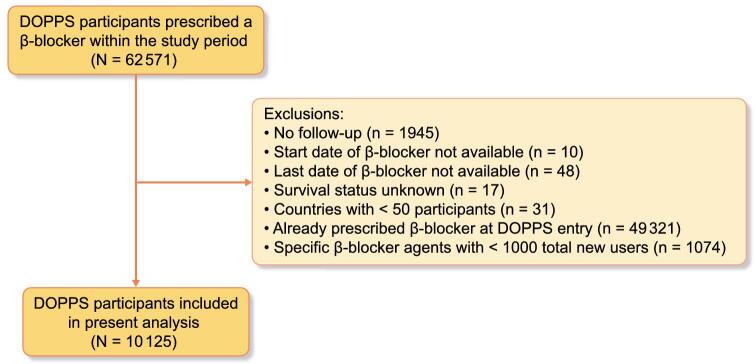
Study cohort flow diagram.

**Figure 2:**
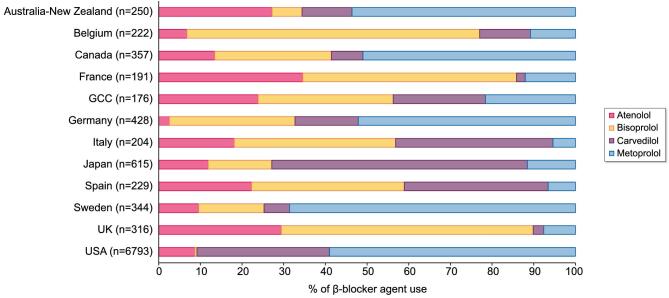
β-blocker agent use by country within DOPPS. GCC, Gulf Cooperation Council (includes Bahrain, Kuwait, Oman, Qatar, Saudi Arabia and the United Arab Emirates).

From a total of 62 571 DOPPS participants prescribed a β-blocker medication within the study window, 10 125 participants met our inclusion/exclusion criteria and were included in the analyses (Fig. 1). Among this population, metoprolol was the most commonly prescribed β-blocker (49%) followed by carvedilol (29%), atenolol (11%) and bisoprolol (11%). The baseline characteristics of the cohort both overall and by β-blocker agent prescribed are displayed in Table 1. The mean (SD) age was 63 (15) years and 57% of participants were male. The majority (67%) of participants were from the USA; however, participants from 17 other countries were also included. Figure 2 displays the breakdown of β-blocker agent use by country. Common comorbidities included hypertension (82%), diabetes mellitus (56%), coronary artery disease (34%), heart failure (28%) and peripheral vascular disease (20%). Hemodialysis characteristics were similar across all β-blocker agents.

**Table 1:** Baseline characteristics of study cohort overall and by individual β-blocker prescribed.

Characteristic	Overall	Atenolol	Bisoprolol	Carvedilol	Metoprolol
Total (*N*)	10 125	1127	1094	2914	4990
Age, mean (SD)	63 (15)	59 (16)	65 (14)	62 (14)	63 (15)
Sex, *N* (%)					
Female	4329 (43)	514 (46)	428 (39)	1174 (40)	2213 (44)
Male	5796 (57)	613 (54)	666 (61)	1740 (60)	2777 (56)
Race, *N* (%)					
Black	2642 (26)	245 (22)	39 (4)	821 (28)	1537 (31)
White	4182 (41)	570 (51)	821 (75)	852 (29)	1939 (39)
Other	3301 (33)	312 (28)	234 (21)	1241 (43)	1514 (30)
Vital signs and anthropometrics, mean (SD)					
Systolic blood pressure, mmHg^a^	147 (24)	149 (25)	140 (24)	148 (23)	147 (24)
Diastolic blood pressure, mmHg^a^	76 (15)	78 (15)	71 (14)	77 (14)	76 (14)
Heart rate, bpm^a^	75 (12)	71 (12)	72 (13)	77 (11)	76 (12)
Body mass index, kg/m^2^	27 (7)	26 (6)	26 (5)	27 (7)	28 (7)
Comorbidities, *N* (%)					
Coronary artery disease	3404 (34)	382 (34)	484 (44)	879 (30)	1659 (33)
Myocardial infarction	915 (9)	83 (7)	178 (16)	206 (7)	448 (9)
Coronary artery bypass graft	622 (6)	69 (6)	104 (10)	165 (6)	284 (6)
Heart failure	2875 (28)	281 (25)	307 (28)	870 (30)	1417 (28)
Stroke	665 (7)	91 (8)	111 (10)	154 (5)	309 (6)
Atrial fibrillation	752 (7)	77 (7)	169 (15)	174 (6)	332 (7)
Pacemaker	210 (2)	15 (1)	42 (4)	59 (2)	94 (2)
Peripheral vascular disease	1998 (20)	230 (20)	330 (30)	523 (18)	915 (18)
Hypertension	8344 (82)	961 (85)	947 (87)	2392 (82)	4044 (81)
Diabetes mellitus	5628 (56)	508 (45)	456 (42)	1775 (61)	2889 (58)
Cancer (not including skin cancer)	846 (8)	85 (8)	138 (13)	211 (7)	412 (8)
Hemodialysis characteristics, median (IQR)					
Vintage, years, median (IQR)	1.9 (0.3–4.7)	1.7 (0.3–4.8)	1.9 (0.4–5.0)	1.9 (0.3–4.6)	1.9 (0.4–4.7)
Sessions per week	3 (3–3)	3 (3–3)	3 (3–3)	3 (3–3)	3 (3–3)
Treatment time, min	229 (208–240)	240 (209–240)	240 (227–240)	227 (207–241)	223 (202–241)
Single-pool Kt/V	1.5 (1.3–1.7)	1.5 (1.3–1.7)	1.5 (1.3–1.7)	1.5 (1.3–1.7)	1.5 (1.4–1.7)

^a^Measured pre-dialysis.

### Mortality risk in the overall cohort

**Figure 3:**
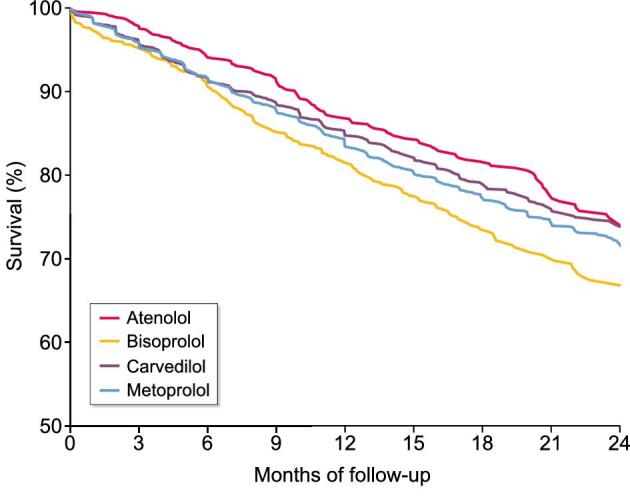
Kaplan–Meier curve for survival by individual β-blocker agent.

**Figure 4:**
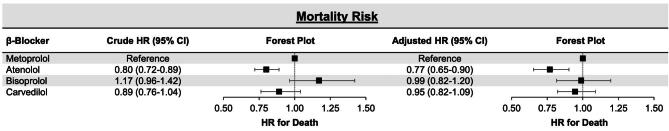
Crude and adjusted mortality risk among maintenance hemodialysis patients by individual β-blocker agent. Adjusted models were used to estimate HRs accounting for the following variables: age, sex, race, body mass index, systolic blood pressure, diastolic blood pressure, dialysis-specific characteristics (vintage, sessions per week, treatment time and single-pool Kt/V), comorbidities [coronary artery disease, myocardial infarction, coronary artery bypass graft surgery, heart failure, atrial fibrillation, pacemaker, stroke, hypertension, peripheral vascular disease, diabetes mellitus and cancer (not including skin cancers)], statin use, clopidogrel use and year of β-blocker initiation.

The crude mortality rates (deaths per 100 person years) for the overall population and by β-blocker agent were as follows: overall 16.6 (95% CI 15.8–17.3), atenolol 13.8 (95% CI 11.8–15.8), bisoprolol 20.1 (95% CI 17.5–22.7), carvedilol 15.3 (95% CI 13.9–16.7) and metoprolol 17.2 (95% CI 16.1–18.3). Figure 3 displays the Kaplan–Meier curve for each individual β-blocker agent. Figure 4 displays the crude and adjusted HR (95% CI) for mortality comparing individual β-blocker agents. In the adjusted models, atenolol [HR 0.77 (95% CI 0.65–0.90)] was associated with a lower mortality risk compared with metoprolol. In contrast, there was no difference in mortality risk between either bisoprolol [HR 0.99 (95% CI 0.82–1.20)] or carvedilol [HR 0.95 (95% CI 0.82–1.09)] and metoprolol.

### Mortality risk based upon prior cardiovascular disease history

**Figure 5:**
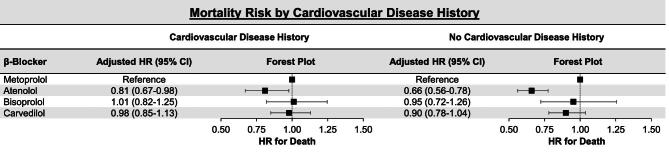
Adjusted mortality risk among maintenance hemodialysis patients by individual β-blocker agent stratified by cardiovascular disease history. Adjusted models were used to estimate HRs accounting for the following variables: age, sex, race, body mass index, systolic blood pressure, diastolic blood pressure, dialysis-specific characteristics (vintage, sessions per week, treatment time and single-pool Kt/V), comorbidities [coronary artery disease, myocardial infarction, coronary artery bypass graft surgery, heart failure, atrial fibrillation, pacemaker, stroke, hypertension, peripheral vascular disease, diabetes mellitus and cancer (not including skin cancers)], statin use, clopidogrel use and year of β-blocker initiation.

Figure 5 displays the results of the stratified analysis based on whether participants had a history of cardiovascular disease [*n* = 5575 (55%)] or not [*n* = 4550 (45%)]. Among participants with a prior history of cardiovascular disease, atenolol [adjusted HR 0.81 (95% CI 0.67–0.98)] was associated with a lower mortality risk compared with metoprolol. In contrast, there was no difference in mortality risk between either bisoprolol [adjusted HR 1.01 (95% CI 0.82–1.25)] or carvedilol [adjusted HR 0.98 (95% CI 0.85–1.13)] and metoprolol. Among participants with no prior history of cardiovascular disease, atenolol [adjusted HR 0.66 (95% CI 0.56–0.78)] was associated with a lower mortality risk compared with metoprolol. There was no difference in mortality risk between either bisoprolol [adjusted HR 0.95 (95% CI 0.72–1.26)] or carvedilol [adjusted HR 0.90 (95% CI 0.78–1.04)] and metoprolol.

### Sensitivity analyses

Sensitivity analyses where blood pressure was not adjusted for within the regression models (Supplementary data, Fig. S1), where participants were stratified by age <65 years or ≥65 years (Supplementary data, Fig. S2), where participants were stratified by whether they had a prior history of heart failure or not (Supplementary data, Fig. S3) and where an “as treated” study design was employed (Supplementary data, Fig. S4) yielded similar results to the primary analysis.

## DISCUSSION

In this large cohort study including 10 125 maintenance hemodialysis patients from 18 countries who were newly prescribed β-blocker medications, we found that atenolol was associated with the lowest mortality risk compared with alternative β-blocker agents. The mortality risk reduction seen with atenolol was similar and consistent for hemodialysis patients with or without a prior history of cardiovascular disease.

To date, clinical trial data for β-blocker use in dialysis patients is extremely limited due to the inherent challenges with conducting a large study in this population. This is best demonstrated by the β-Blocker to LOwer CArdiovascular Dialysis Events (BLOCADE) trial [11]. BLOCADE was designed as a pilot randomized controlled trial across multiple centers in Australia and New Zealand to assess the potential benefit of β-blocker agents in dialysis patients by randomizing 150 dialysis patients to carvedilol versus placebo with 1 year of planned follow-up. Recruitment was extremely limited as after screening 1443 dialysis patients, only 354 were deemed eligible, of which only 91 consented, 72 entered the run-in stage and a mere 49 (3% of those screened) ended up being randomized. The infeasibility of conducting a large clinical trial of β-blockers in dialysis has been postulated to be due to a perceived lack of clinical equipoise surrounding the question of cardiovascular benefit in this population, particularly among patients with heart failure with reduced ejection fraction, post-myocardial infarction or atrial fibrillation, which constitutes roughly half of the dialysis population [12]. To our knowledge, only two small randomized controlled trials dedicated to studying β-blockers in dialysis patients have been performed. One compared carvedilol versus placebo in 114 dialysis patients with cardiomyopathy and found that carvedilol improved left ventricular function and patients’ functional status while also reducing hospitalizations and mortality [8, 9]. A subsequent open-label randomized controlled trial of 200 hemodialysis patients compared atenolol versus lisinopril for reducing left ventricular mass index [10]. The trial was terminated early due to a >2-fold increased rate of serious cardiovascular events in the lisinopril arm.

Given the dearth and infeasibility of robust clinical trial data, real-world observational data provides the strongest evidence supporting β-blocker use in dialysis patients. For instance, a United States Renal Data System study compared all antihypertensive medication categories and found β-blockers to be associated with the highest survival in the hemodialysis population [14]. Similarly, a Taiwanese study of dialysis patients matched 1700 β-blocker users to 1700 non-users and showed reduced mortality among β-blocker users [13]. Similar protective effects with β-blockers in dialysis patients have been shown with reduced mortality following myocardial infarction, reduced mortality with concurrent heart failure and reduced rates of sudden cardiac death [15–17, 27].

With the majority of real-world observational evidence confirming a beneficial effect of β-blockers in the dialysis population, the question of “which (if any) β-blocker agent is preferred for dialysis patients?” has arisen due to heterogeneity in the pharmacologic properties of β-blocker agents. Specifically, cardioselectivity and dialyzability of β-blockers have been studied to explain differential effects in dialysis patients. In terms of cardioselectivity, β-blockers vary widely in their selectivity for β_1_ versus β_2_ receptors [18]. For instance, atenolol, bisoprolol and metoprolol are cardio-(β_1_) selective while carvedilol is non-cardioselective, while also providing α_1_ receptor blockade [28]. A retrospective study of 4938 dialysis patients prescribed β-blockers reported that patients prescribed cardioselective β-blockers experienced a 16% lower all-cause mortality risk compared with patients prescribed non-cardioselective β-blockers [22]. In terms of β-blocker dialyzability, the existing literature is conflicting. A large Canadian study of chronic hemodialysis patients comparing highly versus poorly dialyzable β-blockers found that highly dialyzable β-blockers were associated with an increased mortality risk [20]. In contrast, a large Taiwanese study of chronic hemodialysis patients found that highly dialyzable β-blockers were associated with a reduced mortality risk [21]. The reason behind these discrepant findings may relate to the differential classification of bisoprolol (for which dialyzability is best described as moderate [19]) in these two studies. Notably, bisoprolol accounted for 96% of the poorly dialyzable category in the Canadian study [20] and 59% of the highly dialyzable category in the Taiwanese study [21].

Given this heterogeneity within the β-blocker class of medications, it becomes a challenging and potentially confounded exercise to compare categories of β-blockers based on an isolated pharmacologic factor as multiple other factors may concurrently be in play. To this end, subsequent studies have instead compared individual β-blocker agents head-to-head with one another. For instance, a retrospective study of Medicare-enrolled hemodialysis patients initiated on carvedilol versus metoprolol were compared, with carvedilol users experiencing an 8% higher 1-year all-cause mortality and an 18% higher 1-year cardiovascular mortality [23]. The study also showed that carvedilol users experienced higher rates of intradialytic hypotension, suggesting a potential etiology into the heightened morality risk. A subsequent large study comparing hemodialysis patients initiated on bisoprolol versus carvedilol found bisoprolol to be associated with a 34% lower risk for all-cause mortality along with lower risks for heart failure and ischemic stroke over 2 years of follow-up [24].

The present study extends upon the evidence base behind β-blocker selection in the dialysis population. Using a large well-established international cohort of hemodialysis patients prescribed a variety of different β-blocker agents, we were able to compare multiple β-blockers against one another while accounting for multiple dialysis-specific and patient-based factors. We found that atenolol was associated with the lowest all-cause mortality compared with alternative β-blocker agents. Importantly, the findings of the lowest mortality risk with atenolol was present both in patients with and without a history of cardiovascular disease. While our study was not designed to determine the mechanisms behind why atenolol was associated with lower mortality, it is notable that atenolol is a cardioselective and highly dialyzable β-blocker [19, 28]. One could postulate then that the increased survival benefits with atenolol may therefore be derived from greater selectivity for β_1_ (rather than β_2_) receptors along with increased clearance during dialysis, which may minimize intra-dialytic hypotension and prevent blunting of compensatory sympathetic activity [23]. Interestingly, however, metoprolol is similarly cardioselective and highly dialyzable yet demonstrated an increased mortality risk compared with atenolol. While our study was not designed to determine the mechanism behind this finding, it could potentially be related to several factors. First, in terms of the magnitude of cardioselectivity, atenolol has greater β_1_:β_2_ selectivity (4.7) than that of metoprolol (2.3) [29]. Furthermore, compared with atenolol, metoprolol is more lipophilic and relies more on liver metabolism for clearance [30, 31]. As a result, therapeutic levels are better maintained throughout the inter-dialytic periods for atenolol. In fact, atenolol's half-life in ESKD has been found to be up to 100 h [31]. Further research is required to determine the mechanisms behind why atenolol is associated with a lower mortality risk compared with alternative β-blockers agents; however, these results precisely highlight the heterogeneity amongst β-blockers and the need to compare β-blockers in dialysis patients on an individual basis rather than in categories.

A strength of the current study was the use of a large, representative cohort encompassing data from 18 countries of hemodialysis patients (DOPPS) with heterogeneity in the β-blocker agents prescribed. This facilitated our comparison of multiple β-blocker agents against one another while accounting for patient and dialysis characteristics. We also acknowledge several limitations. First, our results must be interpreted within the context of the study design. Given the observational design, we were able to identify association but not causation. In our analyses, we adjusted for a large number of variables including demographics, vital signs, comorbidities, medications and dialysis-specific characteristics. However, we acknowledge that residual confounding may remain. Second, there was a risk for confounding by indication as certain β-blockers may preferentially be selected based on an individual patient's cardiovascular comorbidities (e.g. carvedilol for heart failure with reduced ejection fraction). However, we not only adjusted for these comorbidities within our analyses, but also stratified our results by presence or absence of any prior cardiovascular disease. Even in patients with no history of cardiovascular disease, the results were consistent. Third, we did not have available data on the specific regimen by which each patient was taking their β-blocker medication. For instance, some patients may have been instructed to skip the medication on dialysis days which we were unable to capture but may have influenced the results. Finally, the results can only be applied to in-center maintenance hemodialysis patients since peritoneal dialysis and home hemodialysis patients were not included in this study.

In conclusion, we observed that atenolol was associated with the lowest mortality risk compared with alternative β-blocker agents in a large international cohort of maintenance hemodialysis patients. This was true for both patients with and without a prior history of cardiovascular disease. These findings will serve to inform clinicians on β-blocker selection for the hemodialysis patient population.

## Supplementary Material

sfae087_Supplemental_File

## Data Availability

The data underlying this article were provided by Arbor Research Collaborative for Health by permission. Data will be shared on request to the corresponding author with permission of Arbor Research Collaborative for Health.
