# Supplementary material for: Comparison of β-blocker agents and mortality in maintenance hemodialysis patients: an international cohort study
Source: Clin Kidney J. 2024 Mar 27;17(5):sfae087. doi: 10.1093/ckj/sfae087 (PMC11181867; doi:10.1093/ckj/sfae087)
Supplement: sfae087_Supplemental_File [file sfae087_Supplemental_File.pdf]

SUPPLEMENTARY MATERIAL

Comparison of β-Blocker Agents and Mortality in Maintenance Hemodialysis Patients: An International Cohort Study

Corey Toye[1], Manish M. Sood[1,2], Ranjeeta Mallick[2], Ayub Akbari[1,2], Brian Bieber[3], Angelo Karaboyas[3], Murilo Guedes[3], and Gregory L. Hundemer[1,2]

Affiliations:
[1] Department of Medicine, Division of Nephrology, University of Ottawa, Ottawa, ON, Canada
[2] Clinical Epidemiology Program, Ottawa Hospital Research Institute, Ottawa, ON, Canada
[3] Arbor Research Collaborative for Health, Ann Arbor, MI, USA

Correspondence To:
Dr. Gregory L. Hundemer
Ottawa Hospital – Riverside Campus
1967 Riverside Drive, Office 5-33
Ottawa, Ontario
Canada
K1H 7W9
Phone: (613) 738-8400
Fax: (613) 738-8337
E-mail: ghundemer@toh.ca

<u>Figure S1:</u> Adjusted mortality risk among hemodialysis patients by individual β-blocker agent; excluding adjustment for blood pressure.

Mortality Risk (No Blood Pressure Adjustment)

Beta Blocker	Adjusted HR (95% CI)	Forest Plot
Metoprolol	Reference	
Atenolol	0.77 (0.66-0.89)	
Bisoprolol	0.99 (0.83-1.19)	
Carvedilol	0.95 (0.82-1.09)	

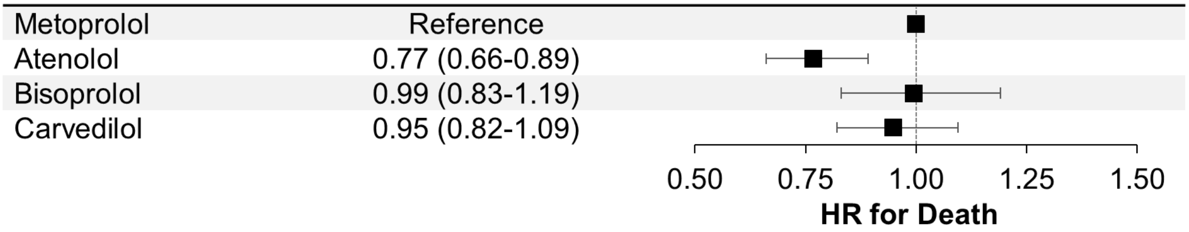

Models accounted for the following variables: age, sex, race, body mass index, dialysis-specific characteristics (vintage, sessions per week, treatment time, and single-pool Kt/V), comorbidities (coronary artery disease, myocardial infarction, coronary artery bypass graft surgery, heart failure, atrial fibrillation, pacemaker, stroke, hypertension, peripheral vascular disease, diabetes mellitus, and cancer [not including skin cancers]), statin use, clopidogrel use, and year of β-blocker initiation.

Abbreviations: CI, confidence interval; HR, hazard ratio.

<u>Figure S2:</u> Adjusted mortality risk among maintenance hemodialysis patients by individual β-blocker agent stratified by age < or ≥65 years.

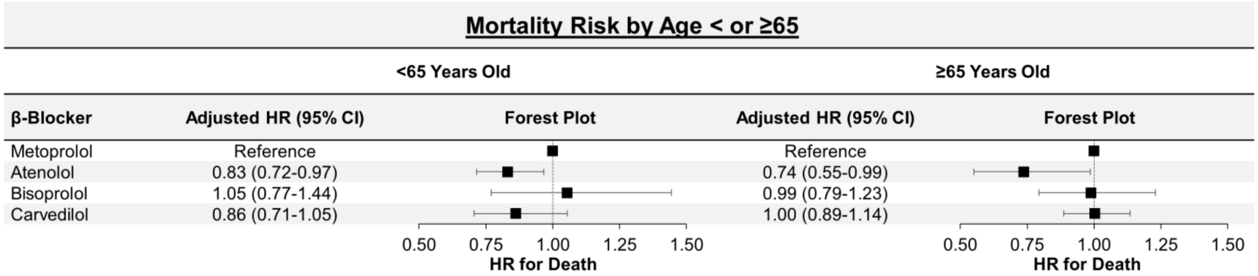

Adjusted models were used to estimate HRs accounting for the following variables: age, sex, race, body mass index, systolic blood pressure, diastolic blood pressure, dialysis-specific characteristics (vintage, sessions per week, treatment time, and single-pool Kt/V), comorbidities (coronary artery disease, myocardial infarction, coronary artery bypass graft surgery, heart failure, atrial fibrillation, pacemaker, stroke, hypertension, peripheral vascular disease, diabetes mellitus, and cancer [not including skin cancers]), statin use, clopidogrel use, and year of β-blocker initiation.

Abbreviations: CI, confidence interval; HR, hazard ratio.

<u>Figure S3:</u> Adjusted mortality risk among maintenance hemodialysis patients by individual β-blocker agent stratified by heart failure history.

<u>Mortality Risk by Heart Failure History</u>

	Heart Failure		No Heart Failure	
**β-Blocker**	**Adjusted HR (95% CI)**	**Forest Plot**	**Adjusted HR (95% CI)**	**Forest Plot**
Metoprolol	Reference		Reference	
Atenolol	0.84 (0.67-1.04)		0.73 (0.61-0.87)	
Bisoprolol	1.10 (0.88-1.39)		0.91 (0.72-1.15)	
Carvedilol	0.97 (0.83-1.12)		0.92 (0.78-1.07)	

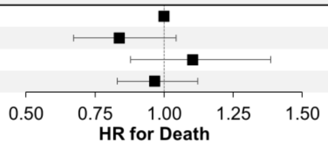

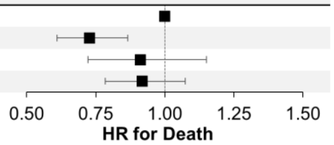

Adjusted models were used to estimate HRs accounting for the following variables: age, sex, race, body mass index, systolic blood pressure, diastolic blood pressure, dialysis-specific characteristics (vintage, sessions per week, treatment time, and single-pool Kt/V), comorbidities (coronary artery disease, myocardial infarction, coronary artery bypass graft surgery, atrial fibrillation, pacemaker, stroke, hypertension, peripheral vascular disease, diabetes mellitus, and cancer [not including skin cancers]), statin use, clopidogrel use, and year of β-blocker initiation.

Abbreviations: CI, confidence interval; HR, hazard ratio.

<u>Figure S4:</u> Adjusted mortality risk among hemodialysis patients by individual β-blocker agent employing an ‘as treated’ analysis.

Mortality Risk (As Treated Analysis)

Beta Blocker	Adjusted HR (95% CI)
Metoprolol	Reference
Atenolol	0.77 (0.66-0.89)
Bisoprolol	0.95 (0.76-1.18)
Carvedilol	0.95 (0.80-1.13)

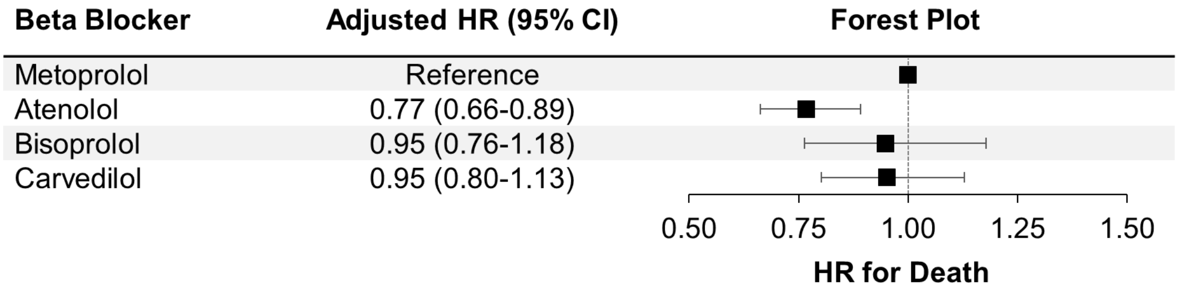

Models accounted for the following variables: age, sex, race, body mass index, dialysis-specific characteristics (vintage, sessions per week, treatment time, and single-pool Kt/V), comorbidities (coronary artery disease, myocardial infarction, coronary artery bypass graft surgery, heart failure, atrial fibrillation, pacemaker, stroke, hypertension, peripheral vascular disease, diabetes mellitus, and cancer [not including skin cancers]), statin use, clopidogrel use, and year of β-blocker initiation.

Abbreviations: CI, confidence interval; HR, hazard ratio.
